# Supplementary material for: Extracellular Vesicles from a Novel Chordoma Cell Line, ARF-8, Promote Tumorigenic Microenvironmental Changes When Incubated with the Parental Cells and with Human Osteoblasts
Source: Int J Mol Sci. 2024 Nov 27;25(23):12731. doi: 10.3390/ijms252312731 (PMC11641215; doi:10.3390/ijms252312731)

Ingenuity Pathway Analysis (IPA) Network Legends, Shapes, and Edge Descriptions

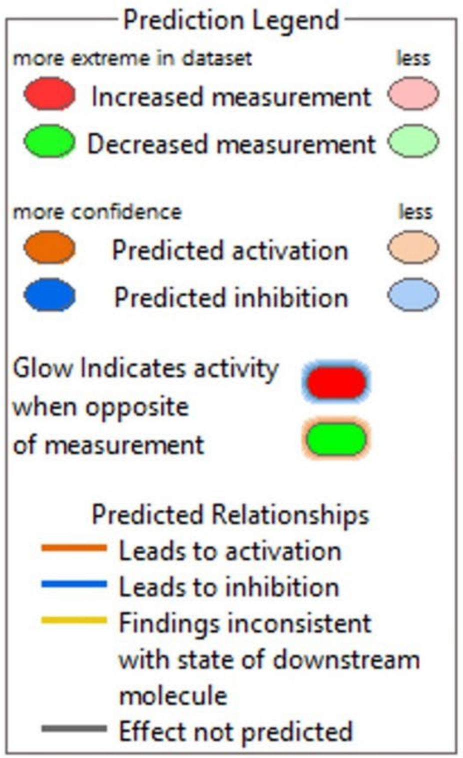

Node Shapes

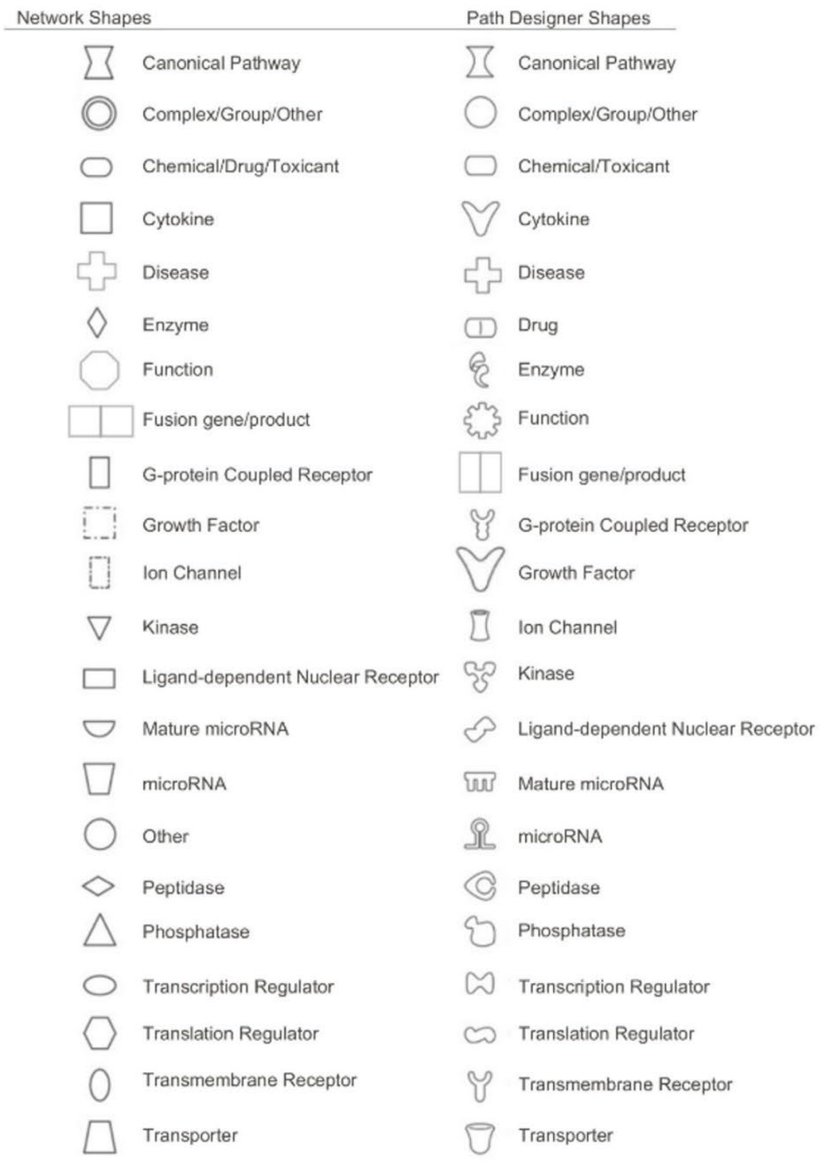

Relationship line descriptions

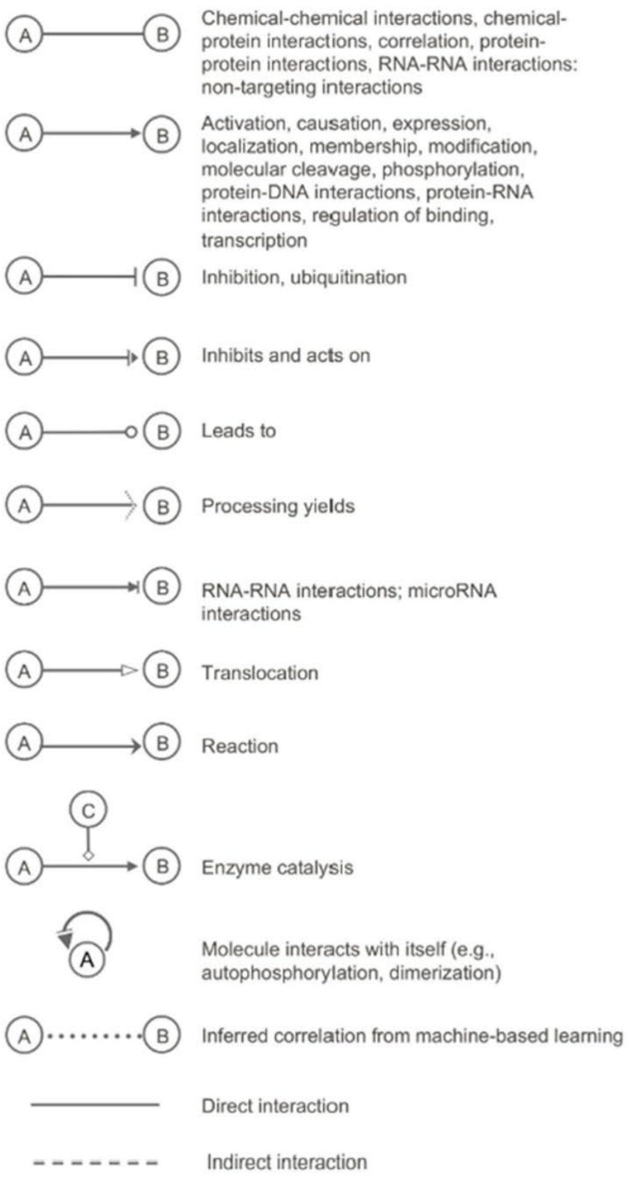

Supplement: Supplementary file 1 [file ijms-25-12731-s001.zip › SUPP FIG S1 IPA.pdf]
