# Supplementary material for: Extracellular Vesicles from a Novel Chordoma Cell Line, ARF-8, Promote Tumorigenic Microenvironmental Changes When Incubated with the Parental Cells and with Human Osteoblasts
Source: Int J Mol Sci. 2024 Nov 27;25(23):12731. doi: 10.3390/ijms252312731 (PMC11641215; doi:10.3390/ijms252312731)
Supplement: Supplementary file 1 [file ijms-25-12731-s001.zip › SUPP FIG S4 ARF8 EMT.pdf]

INGENUITY PATHWAY ANALYSIS

Analysis Name: ARF8 EMT cells&ALL for IPA#2 - 2023-09-10 08:53 AM
Analysis Creation Date: 2023-09-10
Build version: exported
Content version: 94302991 (Release Date: 2023-05-27)

Experiment Metadata

Name	Value

Analysis Settings

Top Canonical Pathways

Name	p-value	Overlap
Pulmonary Fibrosis Idiopathic Signaling Pathway	1.08E-45	17.2 % 56/326
Hepatic Fibrosis / Hepatic Stellate Cell Activation	5.29E-36	20.6 % 40/194
Wound Healing Signaling Pathway	4.34E-30	15.5 % 39/252
Hepatic Fibrosis Signaling Pathway	1.79E-24	9.9 % 42/423
Tumor Microenvironment Pathway	2.12E-24	16.8 % 30/179

(c) 2000-2024 QIAGEN. All rights reserved.

Top Upstream Regulators

Upstream Regulators

Name	p-value	Predicted Activation
TGFB1	1.37E-113	Activated
HRAS	2.47E-70	
KRAS	3.49E-70	Inhibited
beta-estradiol	2.00E-68	Activated
AGT	2.93E-67	Activated

Causal Network

Name	p-value	Predicted Activation
LY2109761	2.33E-100	Inhibited
KLK14	1.13E-99	Activated
TRPS1	3.11E-98	Inhibited
TGFB1	3.21E-97	Activated
ARID1A	4.33E-73	

Top Diseases and Bio Functions

(c) 2000-2024 QIAGEN. All rights reserved.

INGENUITY PATHWAY ANALYSIS

Diseases and Disorders

Name	p-value range	# Molecules
**Cancer**	6.46E-14 - 2.11E-70	318
**Organismal Injury and Abnormalities**	7.26E-14 - 2.11E-70	318
**Reproductive System Disease**	6.46E-14 - 2.33E-48	287
**Tumor Morphology**	3.75E-14 - 2.90E-48	125
**Respiratory Disease**	5.48E-15 - 5.49E-46	240

Molecular and Cellular Functions

Name	p-value range	# Molecules
**Cellular Movement**	3.36E-14 - 8.62E-89	234
**Cellular Growth and Proliferation**	6.21E-14 - 1.13E-54	236
**Cellular Assembly and Organization**	5.13E-14 - 8.11E-52	209
**Cellular Function and Maintenance**	7.69E-14 - 8.11E-52	218
**Cell Death and Survival**	7.69E-14 - 1.30E-51	212

Physiological System Development and Function

Name	p-value range	# Molecules
**Cardiovascular System Development and Function**	6.21E-14 - 2.44E-78	178
**Organismal Development**	6.21E-14 - 2.41E-75	251
**Connective Tissue Development and Function**	6.21E-14 - 1.13E-54	186
**Tissue Development**	6.21E-14 - 1.13E-54	247

(c) 2000-2024 QIAGEN. All rights reserved.

INGENUITY PATHWAY ANALYSIS

**Organismal Survival**	9.35E-20 - 4.94E-53	193

Top Tox Functions

Assays: Clinical Chemistry and Hematology

Name	p-value range	# Molecules
**Increased Levels of Alkaline Phosphatase**	1.32E-02 - 1.27E-10	13
**Decreased Levels of Albumin**	5.19E-02 - 9.09E-06	5
**Increased Levels of LDH**	1.64E-02 - 1.38E-04	5
**Increased Levels of Albumin**	1.48E-01 - 3.57E-04	4
**Increased Levels of Hematocrit**	4.45E-04 - 4.45E-04	7

Cardiotoxicity

Name	p-value range	# Molecules
**Cardiac Dysfunction**	2.24E-01 - 5.80E-34	62
**Cardiac Enlargement**	3.38E-01 - 9.83E-23	58
**Cardiac Fibrosis**	1.36E-01 - 2.03E-18	33
**Cardiac Arrythmia**	5.26E-01 - 7.05E-13	28
**Cardiac Dilation**	3.38E-01 - 1.53E-12	32

Hepatotoxicity

(c) 2000-2024 QIAGEN. All rights reserved.

INGENUITY PATHWAY ANALYSIS

Name	p-value range	# Molecules
**Liver Fibrosis**	1.01E-01 - 8.27E-21	50
**Hepatocellular carcinoma**	3.47E-01 - 9.20E-20	92
**Liver Hyperplasia/Hyperproliferation**	3.47E-01 - 9.20E-20	191
**Liver Proliferation**	3.02E-01 - 1.96E-15	26
**Liver Necrosis/Cell Death**	6.44E-02 - 9.59E-12	25

Nephrotoxicity

Name	p-value range	# Molecules
**Renal Proliferation**	1.01E-01 - 3.38E-13	26
**Glomerular Injury**	4.51E-01 - 4.73E-12	44
**Renal Hydronephrosis**	4.55E-11 - 4.55E-11	14
**Renal Necrosis/Cell Death**	1.59E-01 - 4.66E-11	36
**Renal Damage**	1.35E-01 - 2.44E-10	31

Top Regulator Effect Networks

ID	Regulators	Disease & Functions	Consistency Score
**1**	bleomycin	Migration of carcinoma cell lines	3.474
**2**	YAP1	Cell movement of lung cancer cell lines	3.464
**3**	bleomycin	Epithelial-mesenchymal transition	3.357
**4**	HMG20A	Organismal death	3.336
**5**	aldosterone	Size of body	3.328

(c) 2000-2024 QIAGEN. All rights reserved.

INGENUITY PATHWAY ANALYSIS

Top Networks

ID	Associated Network Functions	Score
1	Organ Development, Reproductive System Development and Function, Organismal Development	38
2	Cell Morphology, Embryonic Development, Hair and Skin Development and Function	31
3	Cellular Movement, Skeletal and Muscular System Development and Function, Cancer	31
4	Dermatological Diseases and Conditions, Inflammatory Disease, Inflammatory Response	31
5	Organismal Injury and Abnormalities, Organismal Survival, Cancer	29

Top Tox Lists

(c) 2000-2024 QIAGEN. All rights reserved.

Name	p-value	Overlap
**Hepatic Fibrosis**	4.45E-38	14.7 % 51/347
**Cardiac Fibrosis**	9.37E-19	9.5 % 33/349
**Genes associated with Chronic Allograft Nephropathy (Human)**	6.17E-18	57.1 % 12/21
**Cardiac Hypertrophy**	1.20E-15	8.0 % 31/387
**Liver Proliferation**	1.04E-14	9.2 % 26/283

Top My Lists

Top My Pathways

Top ML Disease Pathways

Name	p-value	Overlap
**Connective tissue dysplasia**	3.01E-12	23.1 % 12/52
**Dysplasia of skeleton**	2.08E-11	23.4 % 11/47
**Advanced malignant tumor**	8.51E-11	25.0 % 10/40
**Advanced stage tumor**	8.51E-11	25.0 % 10/40
**Corneal neovascularization**	8.80E-11	31.0 % 9/29

Top Analysis-Ready Molecules

Expr Log Ratio

(c) 2000-2024 QIAGEN. All rights reserved.

INGENUITY PATHWAY ANALYSIS

Molecules	Expr. Value	Chart
SULF1	↑ 3.059	
ECM2	↑ 3.016	
FGF2	↑ 3.016	
DNAJB4	↑ 3.000	
ANGPTL4	↑ 2.031	
C1S	↑ 2.000	
COMP	↑ 2.000	
FST	↑ 2.000	
HACL1	↑ 2.000	
LAMA2	↑ 2.000	

Expr Log Ratio

Molecules	Expr. Value	Chart
BMI1	↓ -15.673	
MBNL1	↓ -11.551	
FZD2	↓ -11.551	
FUCA1	↓ -2.710	
COL5A2	↓ -2.419	
MAPKAP1	↓ -0.794	
PLAUR	↓ -0.299	
KLK6	↓ -0.250	
TFPI2	↓ -0.224	
SLIT2	↓ -0.201	

(c) 2000-2024 QIAGEN. All rights reserved.

INGENUITY PATHWAY ANALYSIS
