# Supplementary material for: Extracellular Vesicles from a Novel Chordoma Cell Line, ARF-8, Promote Tumorigenic Microenvironmental Changes When Incubated with the Parental Cells and with Human Osteoblasts
Source: Int J Mol Sci. 2024 Nov 27;25(23):12731. doi: 10.3390/ijms252312731 (PMC11641215; doi:10.3390/ijms252312731)
Supplement: Supplementary file 1 [file ijms-25-12731-s001.zip › supplementary Figures and Tables captions.pdf]

Supplementary Figure S1. Ingenuity Pathway Analysis (IPA) network legends, shapes, and edge descriptions.
Supplementary Figure S2. Ingenuity Pathway Analysis Summary for ARF-8 EV proteomics
Supplementary Figure S3. Ingenuity Pathway Analysis Summary for ARF-8 cell proteomics +/- ARF-8 EVs
Supplementary Figure S4. Ingenuity Pathway Analysis Summary for ARF-8 EMT proteomics

Supplementary Figure S5. Ingenuity Pathway Analysis Summary for hOB cell proteomics +/- ARF-8 EVs
Supplementary Table S1. ARF-8 Extracellular Vesicle proteomics
Supplementary Table S2. ARF-8 cellular proteomics, treated or untreated with ARF-8 EVs
Supplementary Table S3. ARF-8 Epithelial-to-Mesenchymal (EMT) signatures
Supplementary Table S4. Human osteoblast cellular proteomics, treated or untreated with ARF-8 EVs
